# Emphasis on the importance of comprehensive clinical and genetic analysis – spinal muscular atrophy combined with phenylketonuria: A case report

**DOI:** 10.1097/MD.0000000000039076

**Published:** 2024-08-02

**Authors:** Kai Ma, Dong Wang, Wandong Hu, Jie Wang, Chunmei Yu, Zhenqiong Cui, Fangfang Liu

**Affiliations:** a Department of Neurology, Children’s Hospital Affiliated to Shandong University, Jinan, Shandong Province, China; b Pediatric Research Institute, Children’s Hospital Affiliated to Shandong University, Jinan, Shandong Province, China; c Epilepsy Center, Children’s Hospital Affiliated to Shandong University, Jinan, Shandong Province, China; d Department of Ultrasound, Central Hospital Affiliated to Shandong First Medical University, Jinan, Shandong Province, China.

**Keywords:** hyperphenylalaninemia, *PAH*, risdiplam, *SMN1*, spinal muscular atrophy

## Abstract

**Rationale:**

Both spinal muscular atrophy (SMA) and Phenylketonuria (PKU) are caused by biallelic pathogenic mutations. However, there has been no report on case who suffering from both diseases simultaneously. SMA mainly affects the motor function while PKU may have an impact on both the intelligence and motor function. But if only 1 disease is treated while neglecting the other, the treatment effect will be compromised. Here, for the first time, we report a case from China diagnosed with both these diseases and treated properly.

**Patient concerns:**

A boy was admitted to the Children’s Hospital Affiliated to Shandong University (Jinan, China) due to “limb weakness for 19 months” when he was 22 months old. Considering that the child’s motor function development is delayed, we made a comprehensive examinations including inherited metabolic diseases and found a significantly increase of phenylalanine concentration in the blood which indicating PKU. Combined with his typical clinical manifestations of SMA, target capture sequencing followed by Sanger sequencing and multiplex ligation-dependent probe amplification (MLPA) technologies were used for genetic confirmation.

**Diagnoses:**

SMA and PKU was confirmed.

**Interventions:**

The child was treated with risdiplam and low phenylalanine formula immediately when he was diagnosed with both SMA and PKU.

**Outcomes:**

The child showed remarkable improvement in motor function and significant decrease of blood phenylalanine concentration after treatment.

**Lessons:**

To our knowledge, this is the first reported case of SMA combined with PKU. This case expands our understanding of diagnosis for synchronous SMA and PKU and highlights the importance of comprehensive examinations and the utilizing of various genetic testing methods to make an accurate diagnosis of genetic diseases, which may help avoiding the progressive damage caused by certain genetic disease with insidious clinical symptoms.

## 1. Introduction

5q Spinal muscular atrophy (SMA) is a rare autosomal recessive neuromuscular disease caused by homozygous deletion or compound heterozygous mutations in the survival motor neuron (*SMN*)-1 gene (OMIM 253300, 253550, 253400) located on chromosome 5. The neonatal incidence rate of SMA in parts of East Asia is 0.51 to 0.58/10,000,^[1,2]^ with a population carrier rate of 1/50.^[3]^ Biallelic *SMN1* mutations cause SMN protein insufficiency and α-motor neuron apoptosis in the anterior horn of the spinal cord. Clinically, SMA is characterized by symmetrical, progressive muscle weakness and atrophy and is the most common fatal hereditary neuromuscular disease in infants and young children.^[4]^ The intelligence of SMA patients is generally unaffected. Several drugs targeting restoration of SMN expression, namely, nusinersen, risdiplam, and onasemnogene abeparvovec (Zolgensma) have been approved. These drugs have changed the clinical landscape of SMA, prolonging the patient survival time and improving prognosis.

Phenylketonuria (PKU, OMIM #261600) is an autosomal recessive amino acid metabolism disorder caused by compound heterozygous mutations of the phenylalanine hydroxylase (*PAH*) gene on chromosome 12. The incidence rate of PKU in China is 1.22 to 14.55/100,000.^[5,6]^ PAH catalyzes conversion of phenylalanine to tyrosine. In PKU, phenylalanine hydroxylase activity is completely lost or severely decreased, leading to hyperphenylalaninemia. The tyrosine concentration is also reduced, but hypotyrosinemia is not obvious as tyrosine can be obtained through diet. Common clinical manifestations of PKU include intellectual disability, fair skin, blond dry hair, eczema, pungent smelling sweat and urine, and some children may have neurological symptoms such as epilepsy and hyperactivity. Patients with PKU can be treated by long-term dietary restriction therapy. New therapeutic drugs and methods provide new options for patients, including tetrahydrobiopterin cofactors, recombinant phenylalanine lyase, mRNA therapy, and AAV vector gene therapy.^[7]^

Currently, no case of SMA combined with PKU have been reported. Here, we retrospectively analyzed the clinical data of a child with SMA complicated by PKU to aid diagnosis and treatment of similar patients. Our case shows that SMA may be complicated by PKU, suggesting that various detection methods should be used to facilitate differential diagnosis of genetic diseases.

## 2. Case presentation

This study was approved by the Research Ethics Committee of Children’s Hospital Affiliated to Shandong University (approval number QLET-IRB/P-2021090) for genomic studies of rare disorders. All the procedures performed in the work were in accordance with the Declaration of Helsinki. Informed written consent for participation in the study and for the publication of the clinical data was obtained from the child’s guardians.

The male patient was 22 months old when he was admitted to the Children’s Hospital Affiliated to Shandong University (Jinan, China) due to “limb weakness for 19 months.” His motor development was normal after birth. At 2 months of age, he was able to steadily raise his head. At 3 months of age, his limb movement ability began to decline. He could turn over until 4 months but could not sit independently by 6 months of age; his motor function gradually deteriorated thereafter. By the time of consultation, he could not lift his head, turn over, or sit unaided. The child had visited several local hospitals after symptom onset, but a timely diagnosis was not made because of insufficient understanding of the disease. He had a history of recurrent eczema with no specific birth or family history. The parents were not in a consanguineous marriage, and the mother’s physical examination during pregnancy was normal. At hospital admission, the child was conscious and had weak crying, fair skin, unsteady head, decreased limb movement, grade III and II muscle strength of upper and lower limbs, respectively (determined using the Medical Research Council Scale for Muscle Strength), and hypomyotonia. Bilateral biceps, triceps, tendon, and ankle reflexes were not elicited. Cranial magnetic resonance imaging indicated no abnormalities. Electromyography was consistent with neurogenic damage. Routine blood and urine tests were normal (including creatine kinase, thereby excluding Duchenne muscular dystrophy). The further blood metabolic disease screening showed: phenylalanine concentration 870.0 μmol/L (normal range, 24.3–116 μmol/L), tyrosine 50.2 μmol/L (normal range, 25.8–195 μmol/L), and phenylalanine/tyrosine ratio (Phe/Tyr) 17.3 (expected ratio, 0.1–1.5). The phenylalanine concentration and Phe/Tyr ratio were markedly higher than normal. No respiratory or feeding problems were observed. Based upon these clinical manifestations and laboratory examinations, our initial diagnoses were SMA and PKU.

**Figure 1.**
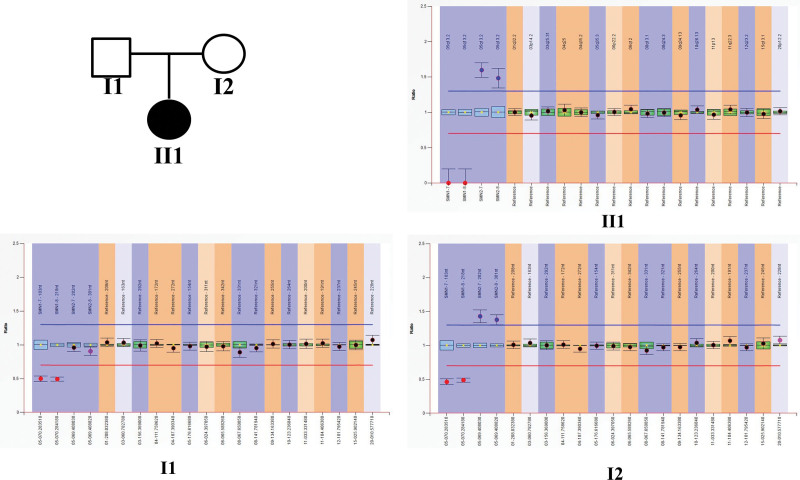
MLPA detection of the *SMN* gene in the child and his parents. Reference samples were part of the MLPA kit. All reference samples showed homozygous expression of exon 7 and 8 of *SMN1* and 2 copies of exon 7 of *SMN2*. The child (II1) had homozygous deletion of exon 7 and 8 of *SMN1* with 3 copies of exon 7 of *SMN2*. The father (I1) and mother (I2) both had heterozygous deletion of exon 7 and 8 of *SMN1* with 2 and 3 copies of exon 7 of *SMN2*, respectively. MLPA: multiplex ligation-dependent probe amplification.

**Figure 2.**
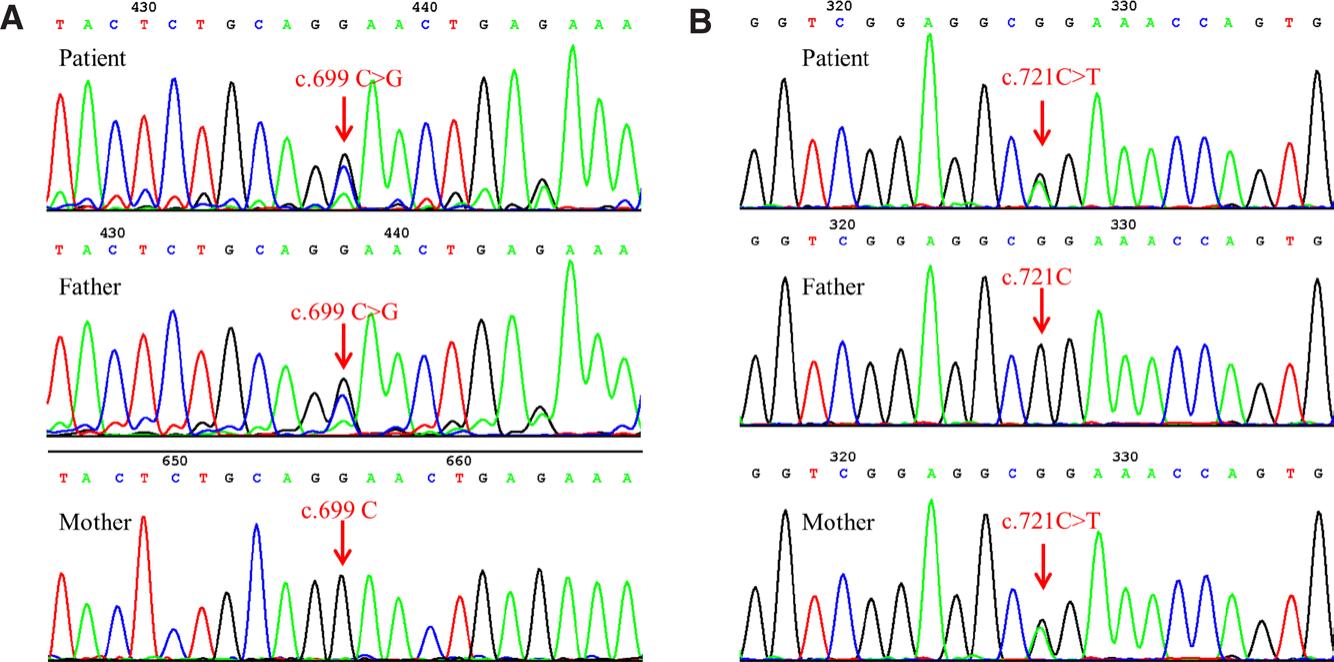
Peak mapping of *PAH* gene mutations in the child and his parents. Electropherograms of the *PAH* gene mutations. Red arrows refer to the mutation site in the patient and the corresponding site in the parents. The patient had c.699C > G (p.Phe233Leu) and c.721C > T (p.Arg241Cys) compound heterozygous mutations. The father had a c.699C > G (p.Phe233Leu) heterozygous mutation. The mother had a c.721C > T (p.Arg241Cys) heterozygous mutation.

To further clarify the diagnoses, the child and his parents were tested for *SMN1* gene variants. The multiplex ligation-dependent probe amplification (MLPA) results showed the child had homozygous deletion of exon 7 and exon 8 in *SMN1* with 3 copies of *SMN2* and his father and mother both had heterozygous deletion of exon 7 and 8 in *SMN1* with 2 and 3 copies of *SMN2*, respectively (Fig. 1). Target capture sequencing followed by Sanger sequencing showed that the child had c.699C > G (p.Phe233Leu) and c.721C > T (p.Arg241Cys) compound heterozygous mutations in the *PAH* gene. The father carried a c.699C > G (p.Phe233Leu) heterozygous mutation, while the mother had a c.721C > T (p.Arg241Cys) heterozygous mutation (Fig. 2). The effect of the c.699C > G (p.Phe233Leu) mutation site on *PAH* protein had not been previously described; therefore, we performed bioinformatic analysis of the identified *PAH* gene mutations. Both variants were located in a highly conserved region in different species and were evaluated in adherence to American College of Medical Genetics and Genomics standards.^[8]^ Accordingly, both variants were determined to be pathogenic, as summarized in Table 1.^[9–14]^

**Table 1:** Evaluation of the variants c.721C > T (p.R241C) and c.699C > G (p.Phe233Leu) based on ACMG criteria.

Variants	Evidence of pathogenicity	Category
c.721C > T (p.R241C)	Strong criterion	PS3: This mutant had 25% normal PAH activity in vitro, and individuals with this homozygous variant had reduced PHE levels as measured by in vivo PHE breath test^[9,10^^]^
	Moderate criterion	PM3_VeryStrong: p.R241C and p.R413P were compound heterozygous for each other in 1 patient, and p.R241C and p.R243Q were compound heterozygous for each other in another patient, and both p.R243Q and p.R413P were pathogenic variants^[11]^
		PM5_Strong: At least 2 different pathogenic amino acid changes (p.R241L and p.R241H) at this variant site (p.R241C) have been reported^[12,13]^
c.699C > G (p.Phe233Leu)	Strong criterion	PS1: The known pathogenic variation of the same amino acid with different nucleotide substitution has been reported
	Moderate criterion	PM2_Supporting: absent from controls in Exome Sequencing Project, 1000 Genomes and Exome Aggregation Consortium
		PM3_Strong: PKU cases with recessive inheritance of this mutation site (c.699C > G) have been reported in the literature database. This variant in the HGMD database is labeled DM. The pathogenicity analysis of this site in ClinVar database was as follows: Pathogenic and PKU
		PM5: 1 pathogenic amino acid changes (p.F233I) at this variant site (p.F233L) have been reported^[14]^
	Supportive criterion	PP3_Moderate: The result of REVEL, a comprehensive protein function prediction software, was deleterious; SIFT, PolyPhen_2, and MutationTaster were deleterious, probably damaging and disease causing, respectively

ACMG = American College of Medical Genetics and Genomics, DM = disease mutation, HGMD = Human Gene Mutation Database, PHE = phenylalanine, PKU = phenylketonuria, PM = moderate criterion, PP = supportive criterion, PS = strong criterion, REVEL = rare exome variant ensemble learner, SIFT = Sorting Intolerant From Tolerant.

Based upon the child’s genetic results, a dual diagnosis of 5q-SMA and PKU was confirmed. The child had developed typical clinical manifestations of SMA and needed immediate treatment. The blood phenylalanine concentration was >360 μmol/L, necessitating dietary treatment.^[15,16]^ After the parents were fully informed of the patient’s condition, they agreed to a treatment plan of risdiplam combined with low phenylalanine formula.

Before treatment, the child’s motor function was evaluated using standard SMA tests. The Children’s Hospital of Philadelphia Infant Test of Neuromuscular Disorders (CHOP INTEND; maximum score, 64) and Hammersmith Infant Neurological Examination Part 2 (HINE-2) scores (maximum score, 26 points) were 23 and 4 points, respectively. The child was also evaluated using the Gesell assessment, scoring 100 points for the adaptive part, indicating a normal intelligence level. Reexamination after 3 months of treatment showed a 6-point increase in the CHOP INTEND score (to 29 points) and a 4-point increase in the HINE-2 score (to 8 points). After 15 months of treatment, the follow-up results showed a 23-point increase in the CHOP INTEND score (to 46 points) and a 5-point increase in the HINE-2 score (to 9 points), respectively. These motor function improvements were clinically significant^[17]^; notably, the patient’s ability to lift his head had improved after treatment. Reexamination of the adaptive part of the Gesell scale maintained a score of 100 points. Retested blood phenylalanine and tyrosine concentrations were 287.2 and 36.1 μmol/L, respectively, with a Phe/Tyr ratio of 7.9. Both the phenylalanine concentration and Phe/Tyr ratio were significantly lower than baseline levels.

## 3. Discussion

In clinical practice, SMA usually refers to 5q-SMA, in which approximately 95% of patients have homozygous deletions of exon 7 of the *SMN1* gene. The remaining 5% of patients have compound heterozygous mutations, with 1 exon 7 deletion and 1 intragenic mutation on the other *SMN1* allele.^[18]^ According to the age of symptom onset and maximum motor function achieved, SMA can be divided into 5 subtypes: type 0 (death typically within 1 month of birth), type I (onset before 6 months, unable to sit independently), type II (onset after 6 months, unable to stand or walk independently), type III (onset after 18 months), and type IV (onset in adulthood). The present patient had symptom onset at 3 months of age and could not sit independently at 6 months. Therefore, he was classified as type I. In the natural course of SMA, the average event-free survival age of type I patients is only 7.3 months.^[19]^ An improved prognosis is achieved with earlier treatment initiation.^[20]^ After 24 months of risdiplam treatment, 44% of patients can achieve the motor function milestone of sitting unaided.^[21]^

The common pathogenic gene of PKU is *PAH*, which has a total length of approximately 90 kb, containing 13 exons and encoding 451 amino acids. Nearly 800 *PAH* gene mutations have been reported worldwide. Without treatment, patients can develop severe intellectual and neurological impairments.^[7]^ Patients receiving long-term dietary restriction therapy can avoid these problems, but if treatment compliance is poor, high phenylalanine levels can cause adverse effects on attention, mood, memory, and executive function in adolescent and adult patients.^[22,23]^ The prognosis of PKU is related to many factors, and treatment timing is important. If treatment is started in the neonatal period, mental and physical development can reach normal levels.^[15]^ Of the 2 *PAH* gene mutations detected in our case, c.721C > T is one of the most common mutations in the Chinese population.^[6,23]^ This mutation results in switching of arginine to cysteine at position 241. Modeling prediction analysis showed that this mutation leads to loss (amino acid position 239) and addition (position 242) of a hydrogen bond. This rearrangement likely affects the spatial structure of the protein. The c.699C > G mutation site is rare^[24]^ and results in a change of phenylalanine to leucine at position 233. Predictive analysis revealed a possible effect of this mutation on enzyme binding but no change in protein structure. The patient’s blood phenylalanine concentration did not exceed 1200 μmol/L and his clinical symptoms were mild, suggesting that the c.699C > G mutation may not lead to complete loss of PAH function.

Currently, more than 10,000 birth defect diseases are known, with an average of 2.8 disease-causing genes per person,^[25]^ which induce heavy burdens on patients, their families, and society. Because of popularization and improvements of gene detection methods, an increasing number of genetic diseases can be identified. The presence of different genetic diseases in the same individual may continue to be discovered. Therefore, for genetic diagnosis of patients with suspected genetic diseases and during prenatal consultations with families, combinations of genetic testing methods should be chosen to detect the abnormal gene copy numbers or/and subtle mutations. For instance, if genetic diagnosis of SMA is made using only the “gold standard,” MLPA detection,^[26]^ subtle mutations of the *SMN1* gene may be missed.^[27]^

It is rare for the same individual to have multiple gene variations with different disease manifestations. No case report of SMA combined with PKU was found in the CNKI full-text, Wanfang, or PubMed databases (from establishment to June 2024). We identified one report of 2 children in the same family who suffered from independent diagnoses of SMA or PKU (CNKI full-text and Wanfang)^[28]^ and 2 case reports of SMA complicated with Duchenne muscular dystrophy (PubMed).^[29,30]^ To our knowledge, this patient is the first reported case worldwide with a diagnosis of SMA combined with PKU, and who has received separate targeted treatments. Treatment with risdiplam and low phenylalanine formula for 3 months and 15 months showed a good effect with no serious adverse events.

The study’s limitations is that: because the efficacy of the combined treatment of risdiplam and low phenylalanine formula requires a longer follow-up analysis, long-term follow-up results for the treatment are lacking in this study. In addition, it is unclear whether the 2 diseases will affect each other.

This study highlights that it is necessary for comprehensive examinations including inherited metabolic diseases and utilizing different genetic analysis methods of patients with congenital defects to avoid the progressive damage caused by the missed diagnosis of some genetic diseases with hidden onset. In addition, timely therapy initiation and intervention for genetic diseases can prevent the malignant progression of the disease.

## Acknowledgments

The authors thank the family for participating in the study. Funding support was provided by the Science and Technology Development Program of Jinan Municipal Health Commission (2021-2-105). The manuscript was designed by KM, DW, and FL. WH, JW, and CY collected the clinical data and executed genetic experiments. KM, DW, and FL wrote the manuscript. All authors contributed to the article and approved the submitted version.

## Author contributions

**Data curation:** Wandong Hu, Zhenqiong Cui.

**Funding acquisition:** Fangfang Liu.

**Investigation:** Kai Ma, Wandong Hu, Jie Wang, Chunmei Yu, Zhenqiong Cui.

**Methodology:** Kai Ma, Jie Wang, Chunmei Yu.

**Resources:** Fangfang Liu.

**Writing – original draft:** Dong Wang, Kai Ma, Fangfang Liu.

**Writing – review & editing:** Dong Wang, Fangfang Liu.
